# Reply to: “Telemedicine for stroke: a Brazilian success story”

**DOI:** 10.1055/s-0046-1820107

**Published:** 2026-07-16

**Authors:** Ícaro Araújo de Sousa, Irapuá Ferreira Ricarte

**Affiliations:** 1 Universidade de São Paulo, Faculdade de Medicina de Ribeirão Preto, Departamento de Neurociências e Ciências do Comportamento, Ribeirão Preto SP, Brazil.; 2 Universidade Federal de São Paulo, Escola Paulista de Medicina, Departamento de Neurologia e Neurocirurgia, São Paulo SP, Brazil.

Dear Editor,

We sincerely would like to thank Dr. Guilherme Nobre Nogueira for his generous and insightful comments on our article, “Telemedicine transforming stroke care in Piauí.” His letter underscores important issues that further enrich the ongoing debate on telestroke implementation in resource-limited regions.

First, we fully agree that cost-effectiveness analyses are essential to evaluate scalability and sustainability. Recent evidence suggests that telestroke programs may represent a cost-effective strategy from social and health-system perspectives. However, most studies to date have been conducted in middle- and high-income countries, 1 which limits our understanding of cost-effectiveness in our real-world context. Interestingly, data from a city in southeastern Brazil indicate that, due to the increased number of thrombolysis procedures, the benefits of telemedicine in the management of ischemic stroke substantially outweigh its costs. 2 As our initial aim was to describe the program's first year, such analyses were not included. Nevertheless, prospective data collection is ongoing and will enable us to assess long-term outcomes, cost-effectiveness, and health system impact in the near future.

Second, regarding the low rate of mechanical thrombectomy (MT; 0.5%), it is important to clarify that this treatment was not available when the stroke care network was initiated in October 2022. Unfortunately, this is not an uncommon scenario. A recent global survey 3 with 526 respondents from 89 countries found that more than 1/5 had no access to MT services, with cost, delayed patient presentation, and, in particular, a shortage of qualified neurointerventionalists identified as the main barriers. Additional challenges include geographic barriers, such as long distances and poor transportation from rural areas; socioeconomic and ethnic inequalities, with high out-of-pocket costs and limited public health system funding; and time-dependent restrictions, in which staff shortages confine availability to daytime hours, preventing 24/7 coverage. 4 In the state of Piauí, the thrombectomy-capable reference center in the capital city of Teresina was inaugurated only in June 2023, midway through the study period. Therefore, the observed rate reflects the short timeframe during which thrombectomy was available and the challenges of transfer logistics and strict eligibility criteria. As statewide referral pathways mature and additional stroke units are established, we expect this number to increase significantly.

Finally, we strongly agree that incorporating innovations such as artificial intelligence-based triage tools, mobile computed tomography strategies, and culturally-adaptive telemedicine platforms is the next natural step. Nevertheless, it is important to emphasize that the immediate priority in Piauí was to establish the very first access to intravenous thrombolysis in the public health system, something that had never been available before. Having achieved this milestone, the program is now moving towards consolidating infrastructure and progressively adopting advanced technologies tailored to local realities.

In conclusion, we greatly appreciate Dr. Nogueira's valuable remarks, which help advance the discussion on equitable and scalable stroke care. Telemedicine has already transformed access to reperfusion therapy in Piauí, demonstrating its feasibility in resource-limited settings. With continued investment in infrastructure, workforce training, and referral networks, this experience has the potential not only to strengthen stroke systems of care locally but also to serve as a model for other underserved regions in Brazil and beyond.
